# Six monthly intravenous zoledronic acid in childhood osteoporosis

**DOI:** 10.1186/1687-9856-2013-S1-P164

**Published:** 2013-10-03

**Authors:** CF Munns, HL Ooi, JN Briody, CT Cowell

**Affiliations:** 1 Institute of Endocrinology & Diabetes, The Sydney Children’s Hospitals Network, Sydney, Australia; 2 Discipline of Paediatrics & Child Health, University of Sydney, Sydney, Australia; 3 Department of Nuclear Medicine, The Sydney Children’s Hospitals Network, Sydney, Australia

## 

Childhood osteoporosis can be treated with intravenous bisphosphonates in order to improve bone mass and density. The aims of this study were to evaluatethe safety and efficacy of six-monthly zoledronic acid (ZA) in children with osteoporosis.

A retrospective cohort study of 27 patients (16 males and 11 females) weretreated with six-monthly ZA (0.05mg/kg/dose) for a minimum of one year.17 patients were immobile, 4 had steroid-induced osteoporosis, 2 had osteogenesis imperfecta and 4 had other diagnoses. 16/27 (59%) had long bone fractures and 12/27 (44.4%) had vertebral wedging at baseline. Mineral homeostasis, bone mineral density by DXA and vertebral morphometry were evaluated at baseline and 1year.

The median age at commencement of treatment was 12.3 years (range 8-15.8). Following the first infusion, 2/27 (7%) and 1/27 (4%) developed asymptomatic hypocalcemia at 48 hours and 72 hours, respectively.A fever above 38°C developed in 14/27 (52%), generalised aches/pains in 13/27 (48%) and nausea in 6/27 (22%).At 1year there was a significant reduction in bone turnover and improvement in bone mineral density (BMD) (see Table 1). Patients with vertebral wedging at baseline showed significant improvement in anterior, middle and posterior vertebral height ratios at 1year. Only one patient fractured after starting ZA. There was normal growth.

**Table 1:** Mineral homeostasis and DXA data at baseline and 1 year

	Baseline	1 year
Calcium (mmol/L)	2.38 (2.35-2.44)	2.36 (2.28-2.42)

Alkaline phosphatase (U/L)	188 (143-271)	148.5 (127.25-205.5)*

Osteocalcin (nmol/L)	7.9 (4.35-11.35)	2.5 (1.1-3.95)*

25-OH-VitD (nmol/L)	75 (67-94)	76 (57.5-86)

Parathyroid hormone (pmol/L)	3.5 (2.3-4.1)	3.7 (2.9-5.4)

Total body aerial BMD Z-score	-0.56 (-1.7 to 0.35)	-0.03 ( -1.13 to 0.86)*

L2-4 aerial BMD Z-score	-1.73 (-2.43 to -0.96)	-0.37 (-1.44 to 0.09)*

Bone mineral content for lean tissue mass Z-score	-1.68 (-2.51 to -0.60)	-0.10 (-0.9 to 1.35)*

Values represent median (interquartile range), asterisk represents p<0.05 compared to baseline

Six monthly ZA was associated with an acute phase reaction to the first dose and improvement in BMD, reduction in bone turnover and improved vertebral shape at 1 year.

